# Supplementary material for: Genome-wide association study identifies a gene responsible for temperature-dependent rice germination
Source: Nat Commun. 2022 Sep 29;13:5665. doi: 10.1038/s41467-022-33318-5 (PMC9523024; doi:10.1038/s41467-022-33318-5)
Supplement: Supplementary file 1 — Supplementary Information [file 41467_2022_33318_MOESM1_ESM.pdf]

Genome-wide association study identifies a gene responsible for temperature-dependent rice germination

Yoshida *et al.*

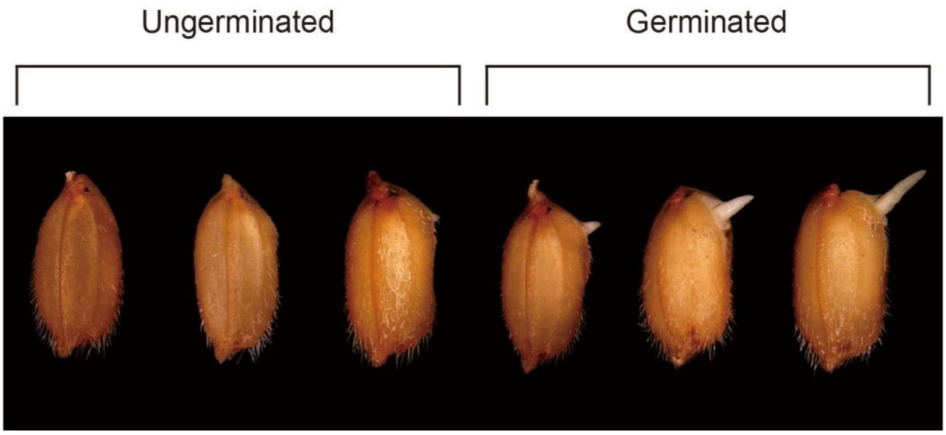

Supplementary Fig. 1. Evaluation of seed germination. Germination was evaluated based on the emergence of a white embryo. The left three seeds were counted as non-germinated, and the right three seeds were counted as germinated.

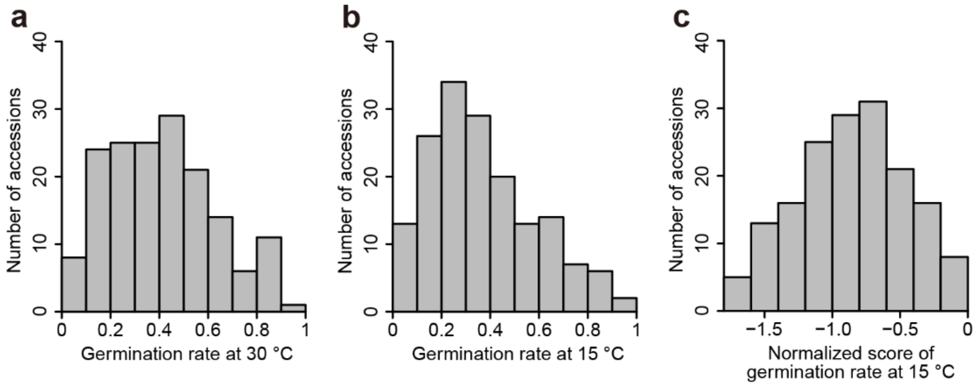

Supplementary Fig. 2. Distribution of seed germination of 164 varieties used in a G × E GWAS. a,b, Germination rates of 164 varieties immersed for 24 h at 30 °C (a) or 96 h at 15 °C (b). Germination rates were calculated as described in the Methods section. **c,** Germination rate for 96 h at 15 °C after box-cox transformation.

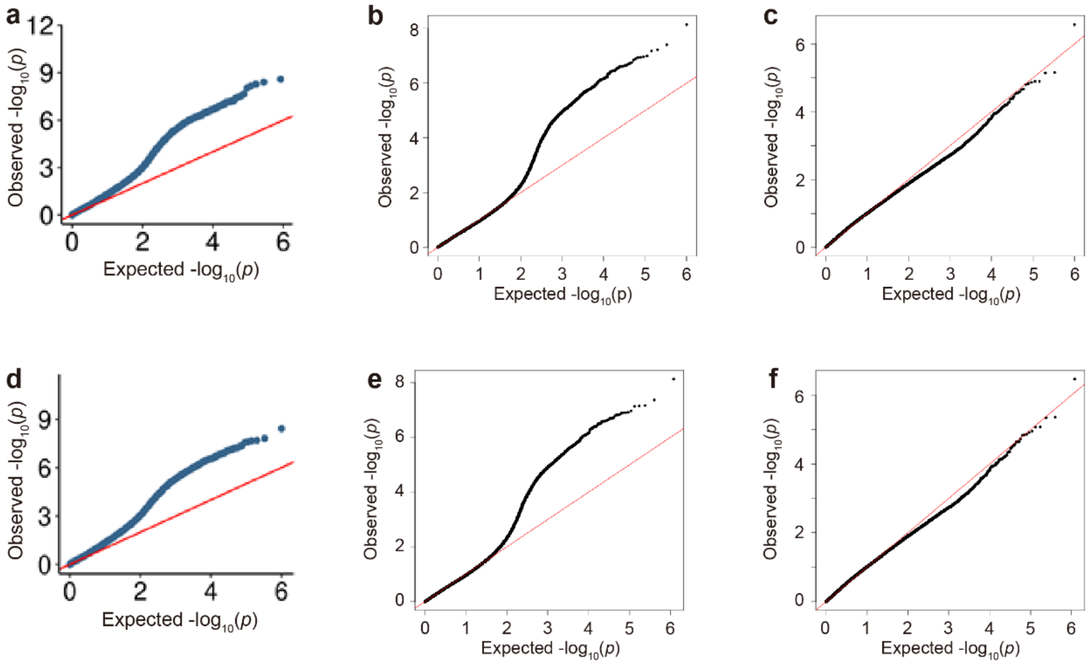

Supplementary Fig. 3. Quantile-quantile plots (Q-Q plots) of the genome-wide association study (GWAS). a–f, Plots correspond to Manhattan plots of GWAS, as shown in Fig. 1a–f, respectively.

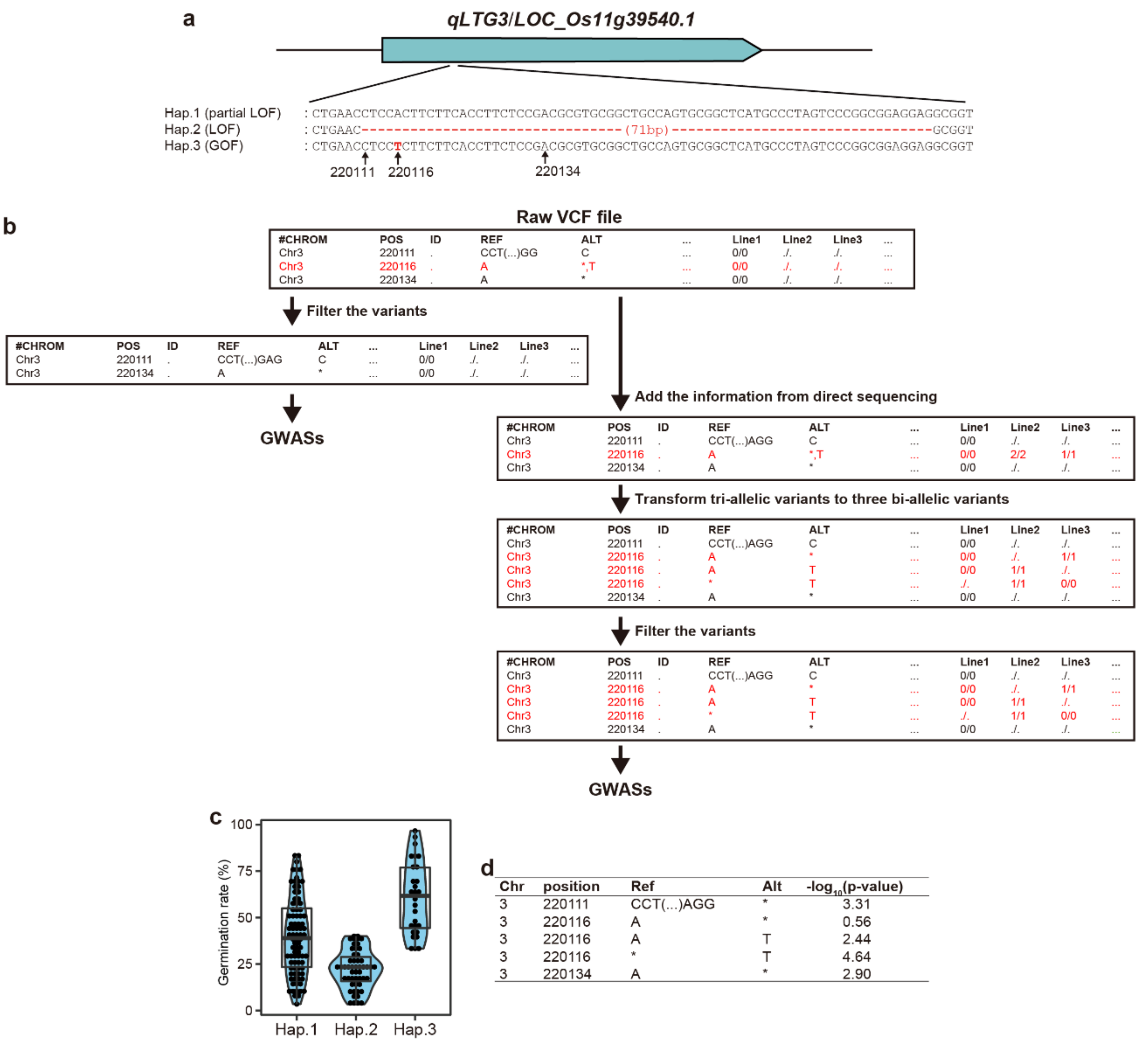

Supplementary Fig. 4. Conversion of functionally different tri-allelic variants of *qLTG3* a, Sequence comparison of three *qLTG3* haplotypes. Haplotype (Hap.)1 is a partial functional allele caused by A220116T substitution (H17L) and Hap.2 is a loss-of-function (LOF) allele caused by a 71 bp deletion, whereas Hap.3 is a gain-of-function (GOF) allele. The numbers indicate positions on chromosome (Chr.) 3. **b,** Workflow of the conversion of genotype data incorporating functionally different tri-allelic variants. The top panel lists the functional polymorphism at 220116 (shown in red), the 5′- and 3′-adjacent ones at 220111 and 220134 (shown in blue), while position 220116 contains tri-allelic polymorphism with A as a reference (REF), * as a deletion, and T as an alternative (ALT). After standard variant call filtering, the tri-allelic polymorphism at 220116 was lost (left side). To avoid this, we transformed the three-allelic variants into three bi-

allelic variants, which contained two REF-ALT pairs and one ALT-ALT pair (right) and filtered the variants, similar to conventional processing. The ALT (LOF)-ALT (GOF) pair showed the maximum difference in the germination rate (Panel c). As Hap.2 contains the 71 bp deletion, we confirmed the presence/absence of this InDel by Sanger sequencing, and added this information to the raw VCF before transformation of three-allelic variants to three variants. **c,** Violin plots of germination rate at 15 °C for 96 h for cultivars that have each haplotype of *qLTG3*. n = 85 (Hap.1), 51 (Hap.2), 28 (Hap.3). Edges of box indicate 25 and 75 percentile points along with medians. Whiskers indicate minima and maxima. **d**, -$\log_{10}$ *P*-values of converted polymorphisms from the genome-wide association study (GWAS) of germination rates at 15 °C for 96 h. The high -$\log_{10}$ *P*-value was observed for the ALT-ALT pair (Hap.2 vs Hap.3), which has not been obtained in other processes, such as prewas system and GATK LeftAlignAndTrimVariants with the 'split-multi-allelics' option, which does not produce the lines representing ALT-ALT pairs.

a

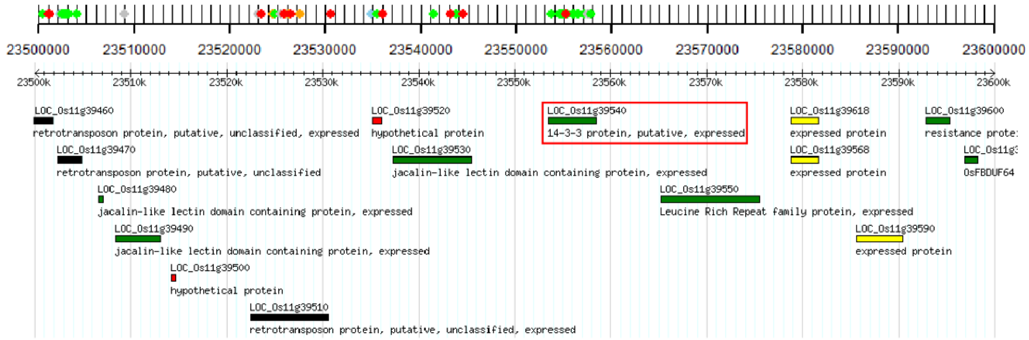

b

Chr	Pos	GeneID	-log10(p-value)	Region	Ref	Alt	Codon	Annotation
11	23543112	LOC_Os11g39530	6.57	CDS	A	T	CAG->CTG	jacalin-like lectin domain containing protein, expressed
11	23544383	LOC_Os11g39530	5.89	CDS	G	A	GTT->ATT	jacalin-like lectin domain containing protein, expressed
11	23544403	LOC_Os11g39530	5.89	CDS	A	T	GAG->GTG	jacalin-like lectin domain containing protein, expressed
11	23544418	LOC_Os11g39530	5.73	CDS	A	C	GAG->GCG	jacalin-like lectin domain containing protein, expressed
11	23555223	LOC_Os11g39540	6.11	CDS	G	TTATG		14-3-3 protein, putative, expressed
11	23611185	LOC_Os11g39640	5.51	CDS	G	C	TTG->TTC	zinc finger, C3HC4 type domain containing protein, expressed
11	23635589	LOC_Os11g39680	5.75	CDS	C	T	GCC->GTC	expressed protein
11	23726086	LOC_Os11g39800	5.75	CDS	G	C	GGA->CGA	expressed protein

Supplementary Fig. 5. Identification of the causal gene for Peak 1. a, A portion of the genome browser view of the candidate region containing GF14h. DNA polymorphisms were categorised into five groups according to their predicted effects on gene functions, from significant (Group I) to insignificant (Group V), as previously reported in Yano *et al.*[1] and shown as red, blue, sky blue, orange, green, and grey dots, respectively. GF14h (LOC_Os11g39540), a causal gene for Peak 1, is shown in red. **b**, List of Group I DNA polymorphisms in the candidate region. The genes annotated to encode “retrotransposon protein” or “hypothetical protein” were omitted. The functional polymorphism of LOC_Os11g39540/GF14h is highlighted in orange.

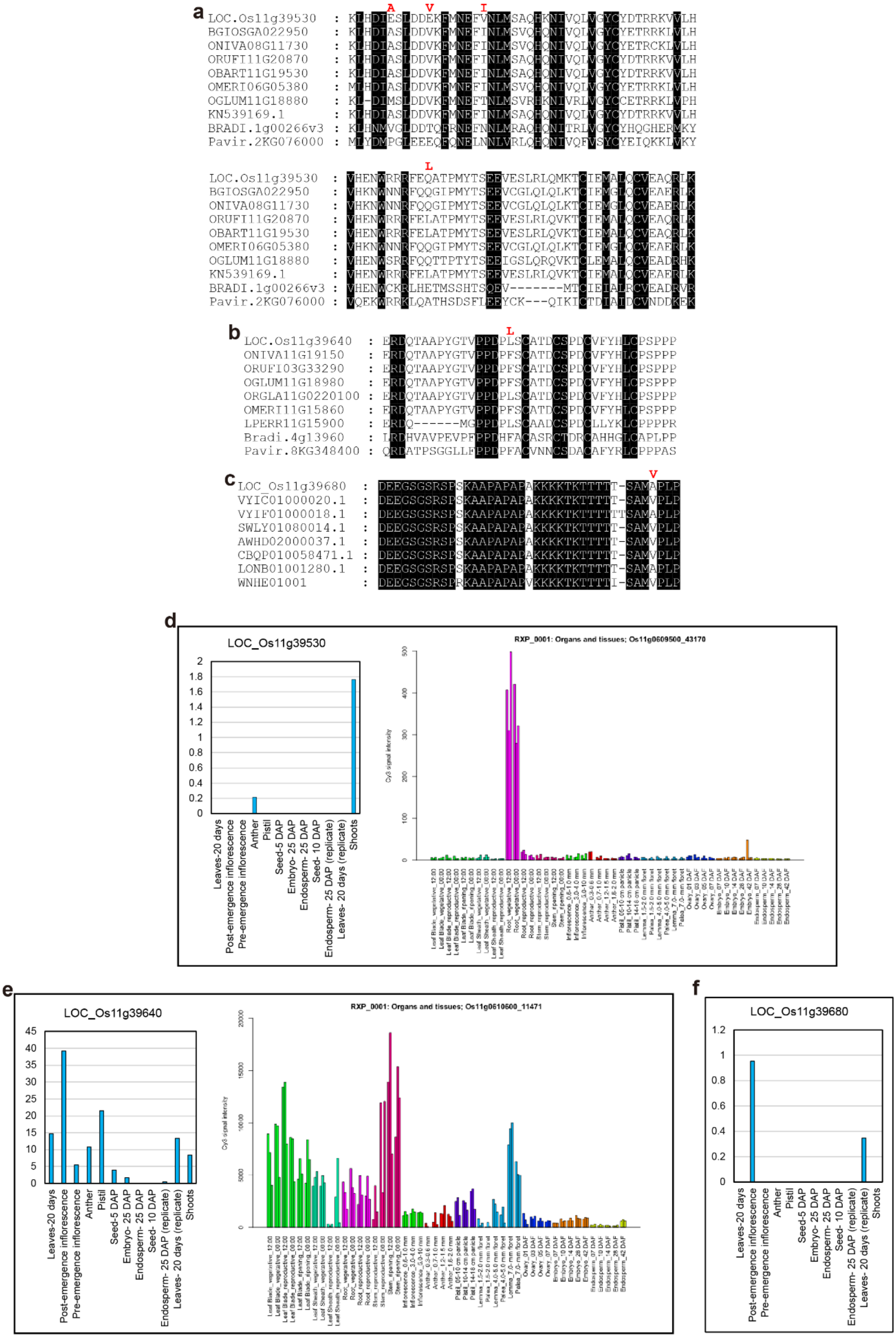

LOC_Os11g39530
RXP_0001: Organs and tissues; Os11g0609500_43170
Cy3 signal intensity
LOC_Os11g39640
RXP_0001: Organs and tissues; Os11g0610600_11471
LOC_Os11g39680
Leaves-20 days
Post-emergence inflorescence
Pre-emergence inflorescence
Anther
Pistil
Seed-5 DAP
Embryo- 25 DAP
Endosperm- 25 DAP
Seed- 10 DAP
Endosperm- 25 DAP (replicate)
Leaves- 20 days (replicate)
Shoots

Supplementary Fig. 6. Amino acid alignment of the candidate protein with its orthologs and public expression data for the genes. a-c, Amino acid alignment of the candidate protein. Exchanged residues based on NPB are indicated above the alignment. These ortholog sequences in Poaceae plants were obtained from Ensemble plants (**a,b**; https://plants.ensembl.org/index.html) or NCBI (**c**; https://blast.ncbi.nlm.nih.gov/Blast.cgi). **d-f**, Public expression data for the candidate genes having their ortholog sequences. The data were obtained from Rice Genome Annotation Project (left; http://rice.uga.edu/) and RiceXpro (right; http://ricexpro.dna.affrc.go.jp/GGEP/index.html). The expression data of LOC_Os11g39680 was not found in RiceXpro. LOC_Os11g39800 is predicted as a gene in Rice Genome Annotation Project but not in RAP-DB (https://rapdb.dna.affrc.go.jp/). Furthermore, Phytozome v13 (https://phytozome-next.jgi.doe.gov/) does not predict any homologs for this gene, and Rice Genome Annotation Project database states that this gene expression is absent in all organs.

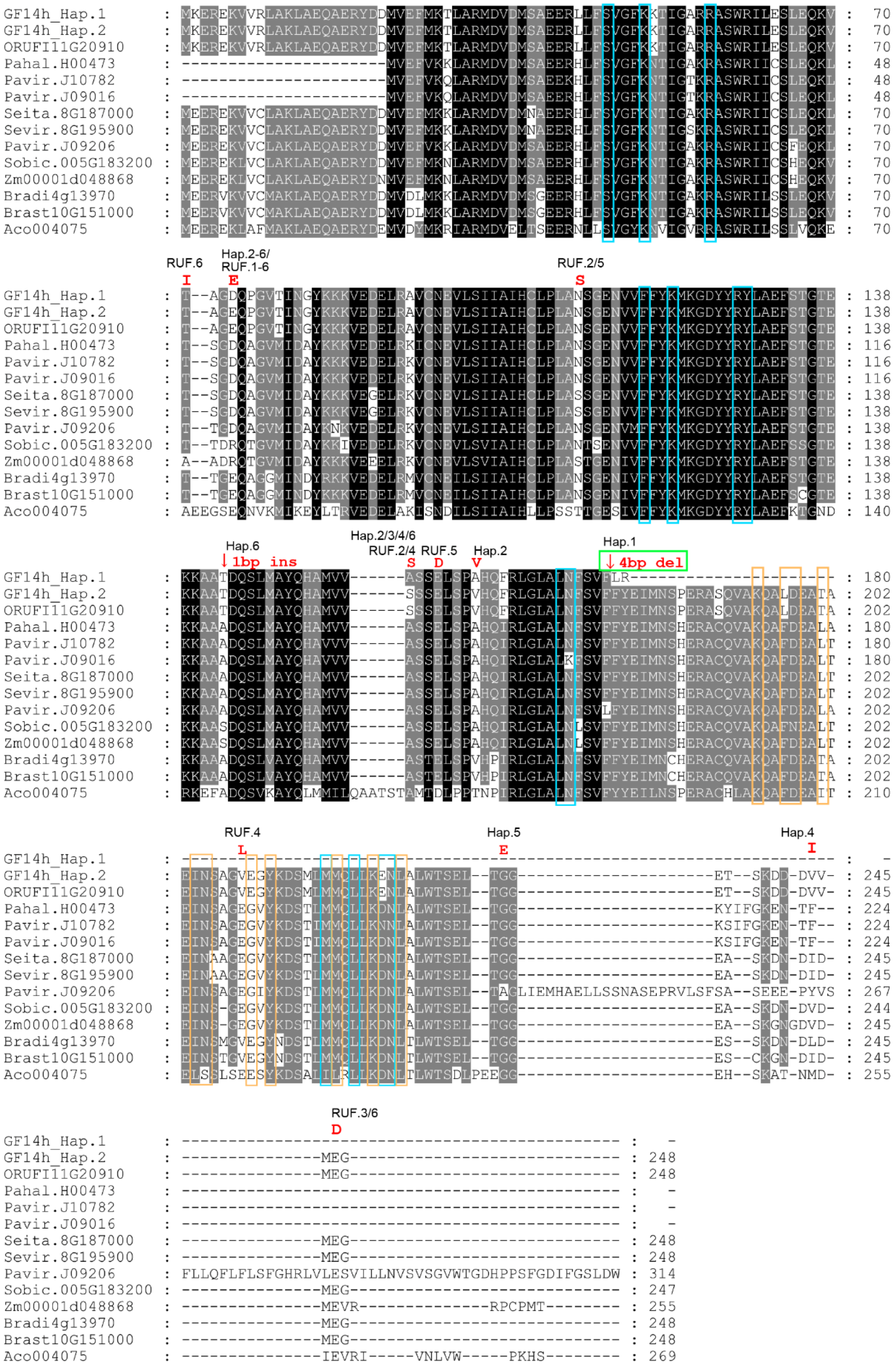
GF14h_Hap.1 : MKEREKVVRLAKLAEQAERYDDMVEFMKTLARMDVDMSAEERLLFSVGFKKTIGARRASWRILESLEQKV : 70
GF14h_Hap.2 : MKEREKVVRLAKLAEQAERYDDMVEFMKTLARMDVDMSAEERLLFSVGFKKTIGARRASWRILESLEQKV : 70
ORUFI11G20910 : MKEREKVVRLAKLAEQAERYDDMVEFMKTLARMDVDMSAEERLLFSVGFKKTIGARRASWRILESLEQKV : 70
Pahal.H00473 : ----------------------MVEFVKKLARMDVDMSAEERHLFSVGFKNTIGAKRASWRIICSLEQKL : 48
Pavir.J10782 : ----------------------MVEFVKQLARMDVDMSAEEKHLFSVGFKNTIGTKRASWRIICSLEQKL : 48
Pavir.J09016 : ----------------------MVEFVKQLARMDVDMSAEERHLFSVGFKNTIGTKRASWRIICSLEQKL : 48
Seita.8G187000 : MEEREKVVCLAKLAEQAERYDDMVEFMKKLARMDVDMNAEERHLFSVGFKNTIGAKRASWRIICSLEQKL : 70
Sevir.8G195900 : MEEREKVVCLAKLAEQAERYDDMVEFMKKLARMDVDMNAEERHLFSVGFKNTIGSKRASWRIICSLEQKL : 70
Pavir.J09206 : MEEREKVVCLAKLAEQAERYDDMVEFVKKLARMDVDMSAEERHLFSVGFKNTIGAKRASWRIICSFEQKL : 70
Sobic.005G183200 : MEEREKIVCLAKLAEQAERYDDMVEFMKNLARMDVDMSAEERHLFSVGFKNTIGAKRASWRIICSHEQKV : 70
Zm00001d048868 : MEEREKIVCLAKLAEQAERYDNMVEFMKNLARMDVDMSAEERHLFSVGFKNTIGAKRASWRIICSHEQKV : 70
Bradi4g13970 : MEERVKVVCMAKLAEQAERYDDMVDLMKKLARMDVDMSGEERHLFSVGFKNTIGAKRASWRILSSLEQKV : 70
Brast10G151000 : MEERVKVVCMAKLAEQAERYDDMVDLMKKLARMDVDMSGEERHLFSVGFKNTIGAKRASWRILSSLEQKV : 70
Aco004075 : MEEREKIAFMAKLAEQAERYDEMVDYMKRIARMDVELTSEERNLLSVGYKNVIGVRRASWRILSSIVQKE : 70
RUF.6
I
Hap.2-6/
RUF.1-6
E
RUF.2/5
S
GF14h_Hap.1 : T--AGDQPGVTINGYKKKVEDELRAVCNEVLSIIAIHCLPLANSGENVVFFYKMKGDYYRYLAEFSTGTE : 138
GF14h_Hap.2 : T--AGEQPGVTINGYKKKVEDELRAVCNEVLSIIAIHCLPLANSGENVVFFYKMKGDYYRYLAEFSTGTE : 138
ORUFI11G20910 : T--AGEQPGVTINGYKKKVEDELRAVCNEVLSIIAIHCLPLANSGENVVFFYKMKGDYYRYLAEFSTGTE : 138
Pahal.H00473 : T--SGDQAGVMIDAYKKKVEDELRKICNEVLSIIAIHCLPLANSGENVVFFYKMKGDYYRYLAEFSTGTE : 116
Pavir.J10782 : T--SGDQAGVMIDAYKKKVEDELRKVCNEVLSIIAIHCLPLANSGENVVFFYKMKGDYYRYLAEFSTGTE : 116
Pavir.J09016 : T--SGDQAGVMIDAYKKKVEDELRKVCNEVLSIIAIHCLPLANSGENVVFFYKMKGDYYRYLAEFSTGTE : 116
Seita.8G187000 : T--SGDQAGVMIDAYKKKVEGELRKVCNEVLSIIAIHCLPLASSGENVVFFYKMKGDYYRYLAEFSTGTE : 138
Sevir.8G195900 : T--SGDQAGVMIDAYKKKVEGELRKVCNEVLSIIAIHCLPLASSGENVVFFYKMKGDYYRYLAEFSTGTE : 138
Pavir.J09206 : T--TGDQAGVMIDAYKNKVEDELRKVCNEVLSIIAIHCLPLANSGENVMFFYKMKGDYYRYLAEFSTGTE : 138
Sobic.005G183200 : T--TDRQTGVMIDAYKKIVEDELRKVCNEVLSIIAIHCLPLANTSENVVFFYKMKGDYYRYLAEFSSGTE : 138
Zm00001d048868 : A--ADRQTGVMIDAYKKKVEEELRKVCNEVLSVIAIHCLPLASTGENIVFFYKMKGDYYRYLAEFSTGTE : 138
Bradi4g13970 : T--TGEQAGGMINDYRKKVEDELRMVCNEILSIIAIHCLPLANSGENIVFFYKMKGDYYRYLAEFSTGTE : 138
Brast10G151000 : T--TGEQAGGMINDYKKKVEDELRMVCNEILSIIAIHCLPLANSGENIVFFYKMKGDYYRYLAEFSCGTE : 138
Aco004075 : AEEGSEQNVKMIKEYLTRVEDELAKISNDILSIIAIHLLPSSTTGESIVFFYKMKGDYYRYLAEFKTGND : 140
Hap.6
↓1bp ins
Hap.2/3/4/6
RUF.2/4
S
RUF.5
D
Hap.2
V
Hap.1
↓4bp del
GF14h_Hap.1 : KKAATDQSLMAYQHAMVV------ASSELSPAHQFRLGLALNFSVFLR------------------ : 180
GF14h_Hap.2 : KKAATDQSLMAYQHAMVV------SSSELSPVHQFRLGLALNFSVFFYEIMNSPERASQVAKQALDEATA : 202
ORUFI11G20910 : KKAATDQSLMAYQHAMVV------SSSELSPVHQFRLGLALNFSVFFYEIMNSPERASQVAKQALDEATA : 202
Pahal.H00473 : KKAAADQSLMAYQHAMVV------ASSELSPAHQIRLGLALNFSVFFYEIMNSHERACQVAKQAFDEALA : 180
Pavir.J10782 : KKAAADQSLMAYQHAVVV------ASSELSPAHQIRLGLALNFSVFFYEIMNSHERACQVAKQAFDEALT : 180
Pavir.J09016 : KKAAADQSLMAYQHAVVV------ASSELSPAHQIRLGLALKFSVFFYEIMNSHERACQVAKQAFDEALT : 180
Seita.8G187000 : KKAAADQSLMAYQHAMVV------ASSELSPAHQIRLGLALNFSVFFYEIMNSHERACQVAKQAFDEALT : 202
Sevir.8G195900 : KKAAADQSLMAYQHAMVV------ASSELSPAHQIRLGLALNFSVFFYEIMNSHERACQVAKQAFDEALT : 202
Pavir.J09206 : KKAAADQSLMAYQHAMVV------ASSELSPAHQIRLGLALNFSVLFYEIMNSHERACQVAKQAFDEALA : 202
Sobic.005G183200 : KKAASDQSLMAYQHAMVV------ASSELSPAHQIRLGLALNLSVFFYEIMNSHERACQVAKQAFNEALT : 202
Zm00001d048868 : KKAASDQSLMAYQHAMVV------ASSELSPAHQIRLGLALNLSVFFYEIMNSHERACQVAKQAFDEALT : 202
Bradi4g13970 : KKAAADQSLVAYQHAMVV------ASTELSPVHPIRLGLALNFSVFFYEIMNCHERACQVAKQAFDEATA : 202
Brast10G151000 : KKAAADQSLVAYQHAMVV------ASTELSPVHPIRLGLALNFSVFFYEIMNCHERACQVAKQAFDEATA : 202
Aco004075 : RKEFADQSVKAYQLMMILQAATSTAMTDLPETNPIRLGLALNFSVFYYEILNSPERACHLAKQAFDEAIT : 210
RUF.4
L
Hap.5
E
Hap.4
I
GF14h_Hap.1 : --- : -
GF14h_Hap.2 : EINSAGVEGYKDSMLMMQLLKENLALWTSEL--TGG---------------------ET--SKDD-DVV- : 245
ORUFI11G20910 : EINSAGVEGYKDSMLMMQLLKENLALWTSEL--TGG---------------------ET--SKDD-DVV- : 245
Pahal.H00473 : EINSAGEGVYKDSTLMMQLLKDNLALWTSEL--TGG---------------------KYIFGKEN-TF-- : 224
Pavir.J10782 : EINSAGEGVYKDSTLMMQLLKNNLALWTSEL--TGG---------------------KSIFGKEN-TF-- : 224
Pavir.J09016 : EINSAGEGVYKDSTIMMQLLKDNLALWTSEL--TGG---------------------KSIFGKEN-TF-- : 224
Seita.8G187000 : EINAAGEGVYKDSTLMMQLLKDNLALWTSEL--TGG---------------------EA--SKDN-DID- : 245
Sevir.8G195900 : EINAAGEGVYKDSTLMMQLLKDNLALWTSEL--TGG---------------------EA--SKDN-DID- : 245
Pavir.J09206 : EINSAGEGIYKDSTLMMQLLKDNLALWTSEL--TACLIEMHAELLSSNASEPRVLSFSA--SEEE-PYVS : 267
Sobic.005G183200 : EINS-GEGVYKDSTLMMQLLKDNLALWTSEL--TGG---------------------EA--SKDN-DVD- : 244
Zm00001d048868 : EINS-GEGVYKDSTLMMQLLKDNLALWTSEL--TGG---------------------EA--SKGNGDVD- : 245
Bradi4g13970 : EINSMGVEGYNDSTLMMQLLKDNLTLWTSEL--TGG---------------------ES--SKDN-DLD- : 245
Brast10G151000 : EINSTGVEGYNDSTLMMQLLKDNLTLWTSEL--TGG---------------------ES--CKGN-DID- : 245
Aco004075 : ELSSLSEESYKDSALILRLLKDNLTLWTSDLPEEGG---------------------EH--SKAT-NMD- : 255
RUF.3/6
D
GF14h_Hap.1 : -- : -
GF14h_Hap.2 : ---------------MEG------------------------------ : 248
ORUFI11G20910 : ---------------MEG------------------------------ : 248
Pahal.H00473 : -- : -
Pavir.J10782 : -- : -
Pavir.J09016 : -- : -
Seita.8G187000 : ---------------MEG------------------------------ : 248
Sevir.8G195900 : ---------------MEG------------------------------ : 248
Pavir.J09206 : FLLQFLFLFSFGHRLVLESVILLNVSVSGVWTGDHPPSFGDIFGSLDW : 314
Sobic.005G183200 : ---------------MEG------------------------------ : 247
Zm00001d048868 : ---------------MEVR--------------RPCPMT-------- : 255
Bradi4g13970 : ---------------MEG------------------------------ : 248
Brast10G151000 : ---------------MEG------------------------------ : 248
Aco004075 : ---------------IEVRI-----VNLVW-----PKHS-------- : 269

Supplementary Fig. 7. Amino acid alignment of the GF14h$^{Hap.1}$ and GF14h$^{Hap.2}$ protein with its orthologs in monocot plants. Exchanged residues in various haplotypes based on GF14h$^{Hap.1}$are indicated above the alignment. Haplotype (Hap.)1–3 were found in the genome-wide association study (GWAS) panel, Hap.4–6 in the 3K panel, and RUF.3–6 in the *Oryza rufipogon* panel. The orthologues of GF14h from *O. rufipogon*, *Brachypodium distachyon*, *Brachypodium stacei*, *Panicum hallii*, *Panicum virgatum*, *Setaria italica*, *Setaria viridis*, *Sorghum bicolor*, *Zea mays*, and *Ananas comosus* were collected by BLAST-P on Phytozome (https://phytozome-next.jgi.doe.gov/). Residues in orange boxes correspond to those identified at Hd3a-binding site[2]. Residues in blue boxes correspond to those interacting with FD1[2].

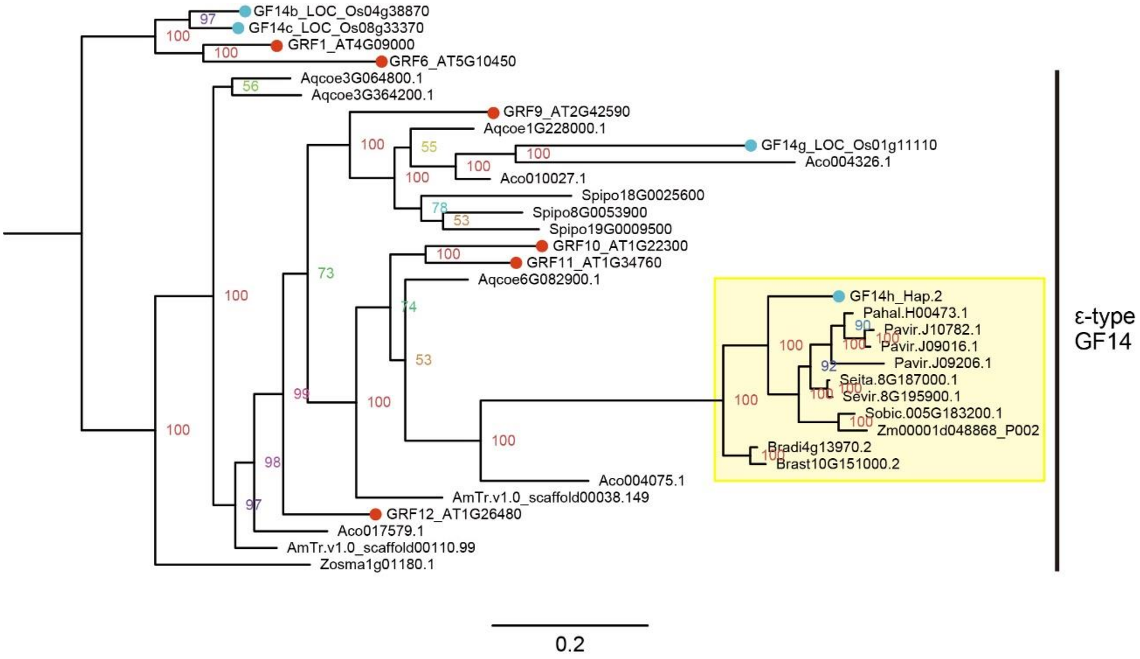

Supplementary Fig. 8. Phylogenetic analysis of ε-type GF14. The clade including GF14h is shown in yellow. Non-ε-type GF14 proteins were used as outgroups (highlighted in blue). Red and blue dots represent *Arabidopsis thaliana* and *Oryza sativa,* respectively. Source data are provided as a Source Data file.

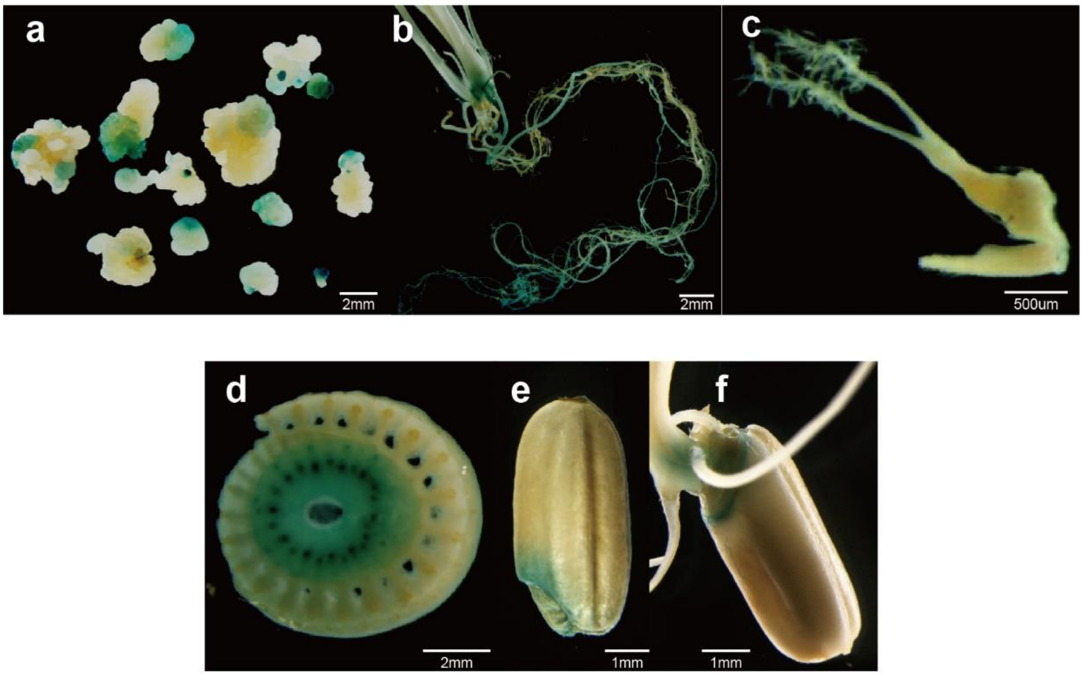

Supplementary Fig. 9. GUS staining of various organs of the pGF14h$^{Hap.2}$::GUS transgenic plants. a, Callus; b**,** root; c**,** anther; **d,** stem; e**,** seed; and **f,** young seedlings. Each experiment was repeated independently for at least 3 times with similar results.

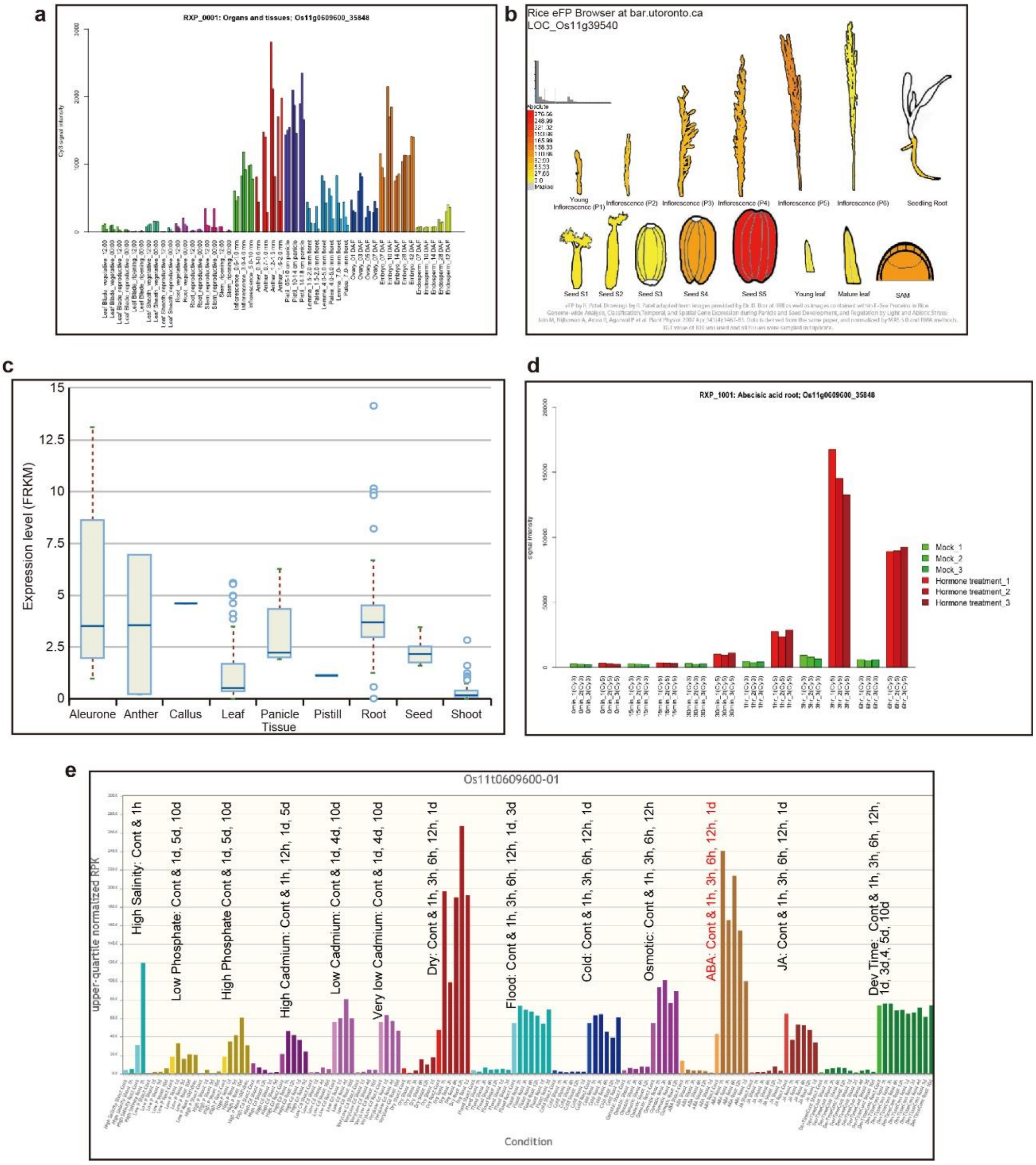

Supplementary Fig. 10. Public expression data for GF14h (LOC_Os11g39540/Os11g0609600). The data were obtained from RiceXpro (**a, d**; http://ricexpro.dna.affrc.go.jp/GGEP/index.html), ePlant (**b**, http://bar.utoronto.ca/eplant_rice/), IC4R (**c**; http://expression.ic4r.org/), and TENOR (**e**; https://tenor.dna.affrc.go.jp/).

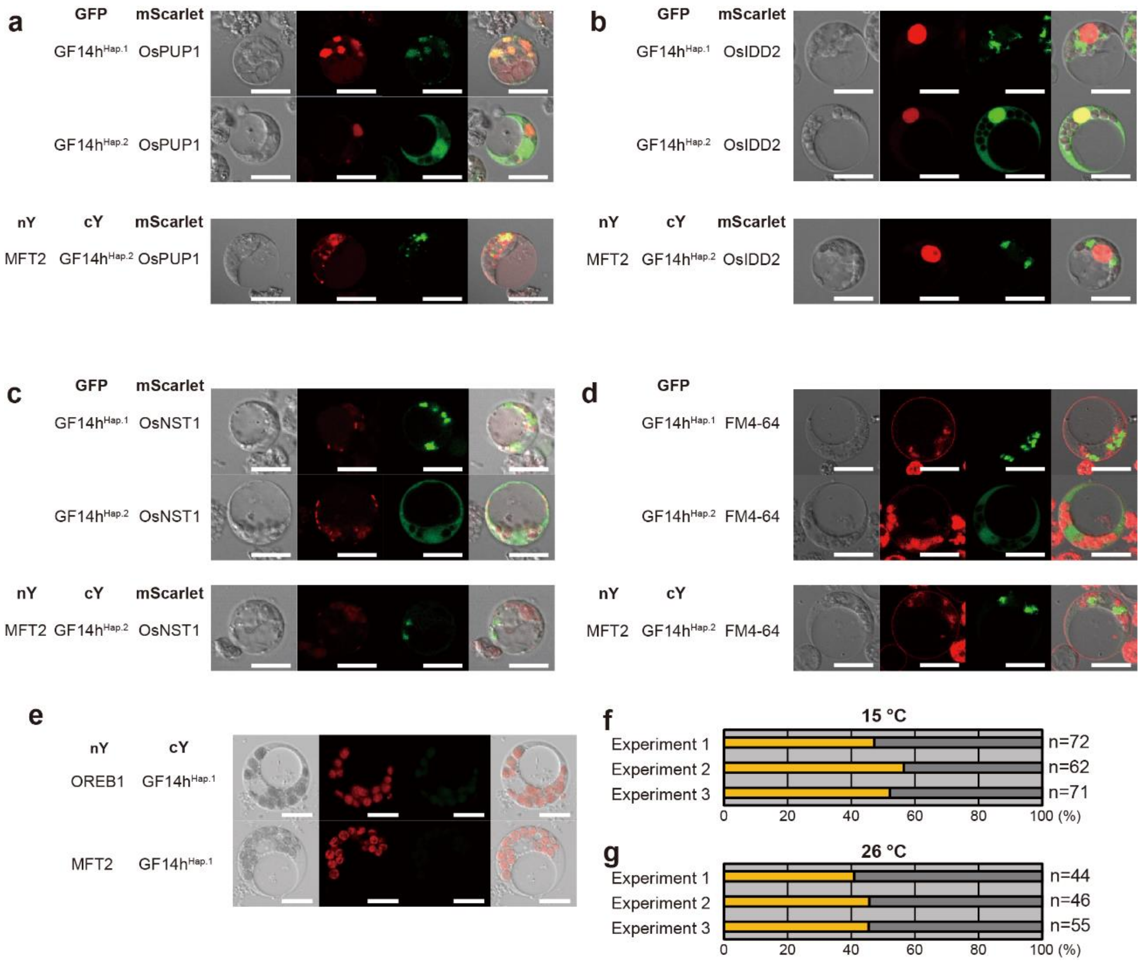

Supplementary Fig. 11. Cellular property of GF14h. a–d, Subcellular localisation of GF14h$^{\mathrm{Hap.1}}$, GF14h$^{\mathrm{Hap.2}}$, and GF14h$^{\mathrm{Hap.2}}$-MFT2 complex. The purine permease, OsPUP1 (**a**), the zinc finger-type transcription factor, OsIDD2 (**b**), the nucleotide sugar transporters, OsNST1 (**c**) and FM4-64 (**d**) were used as markers of endoplasmic reticulum, nucleus, golgi apparatus and plasma membrane, respectively. Individual and merged images of GFP or YFP (green) and mScarlet, or FM4-64 and chlorophyll autofluorescence (red) as well as differential interference contrast images of protoplasts are shown. Scale bars, 10 μm. **e,** BiFC assay for the interaction of GF14h$^{\mathrm{Hap.1}}$-OREB1 and GF14h$^{\mathrm{Hap.1}}$-MFT2. For **a-e**, each experiment was repeated independently for at least 3 times with similar results. Scale bars, 10 μm. **f, g,** Quantification of BiFC assay for the GF14h$^{\mathrm{Hap.2}}$-OREB1 interaction at 15 °C (**f**) or 26 °C (**g**). Source data are provided as a Source Data file.

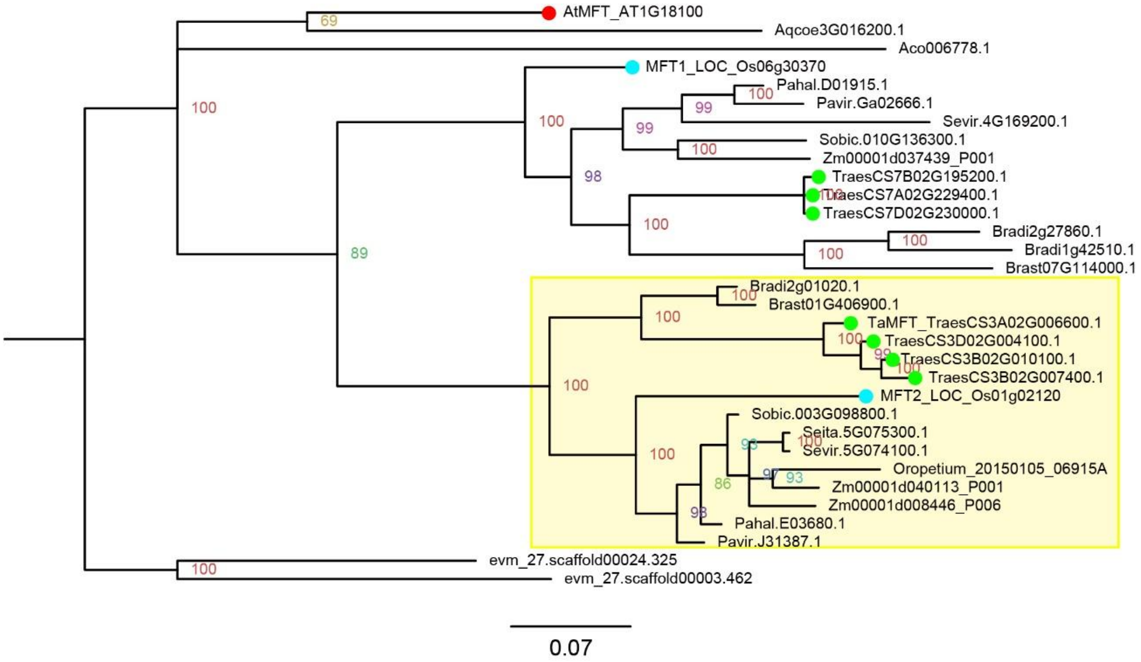

Supplementary Fig. 12. Phylogenetic analysis of MFT2. *Amborella trichopoda* proteins closest to rice MFT2 in the BLAST-P search were used as outgroups (highlighted in blue). The clade containing MFT2 is boxed in yellow. Red, blue, and green dots represent *Arabidopsis thaliana, Oryza sativa,*and *Triticum aestivum*, respectively. Source data are provided as a Source Data file.

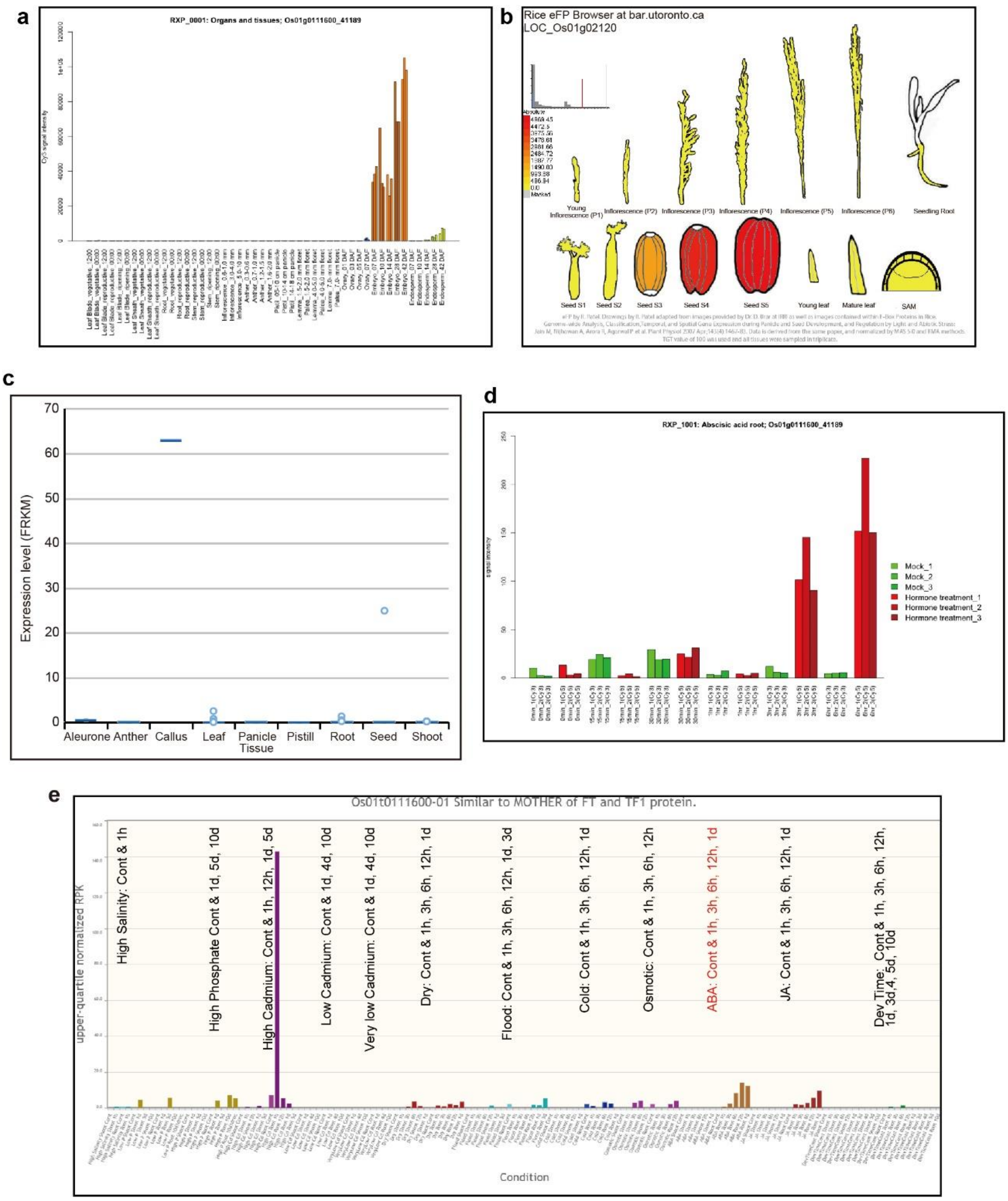

Supplementary Fig. 13. Public expression data for MFT2 (LOC_Os01g02120/ Os01g0111600). The data were obtained from RiceXpro (**a**, **d**; http://ricexpro.dna.affrc.go.jp/GGEP/index.html), ePlant (**b**, http://bar.utoronto.ca/eplant_rice/), IC4R (**c**; http://expression.ic4r.org/), and TENOR (**e**; https://tenor.dna.affrc.go.jp/).

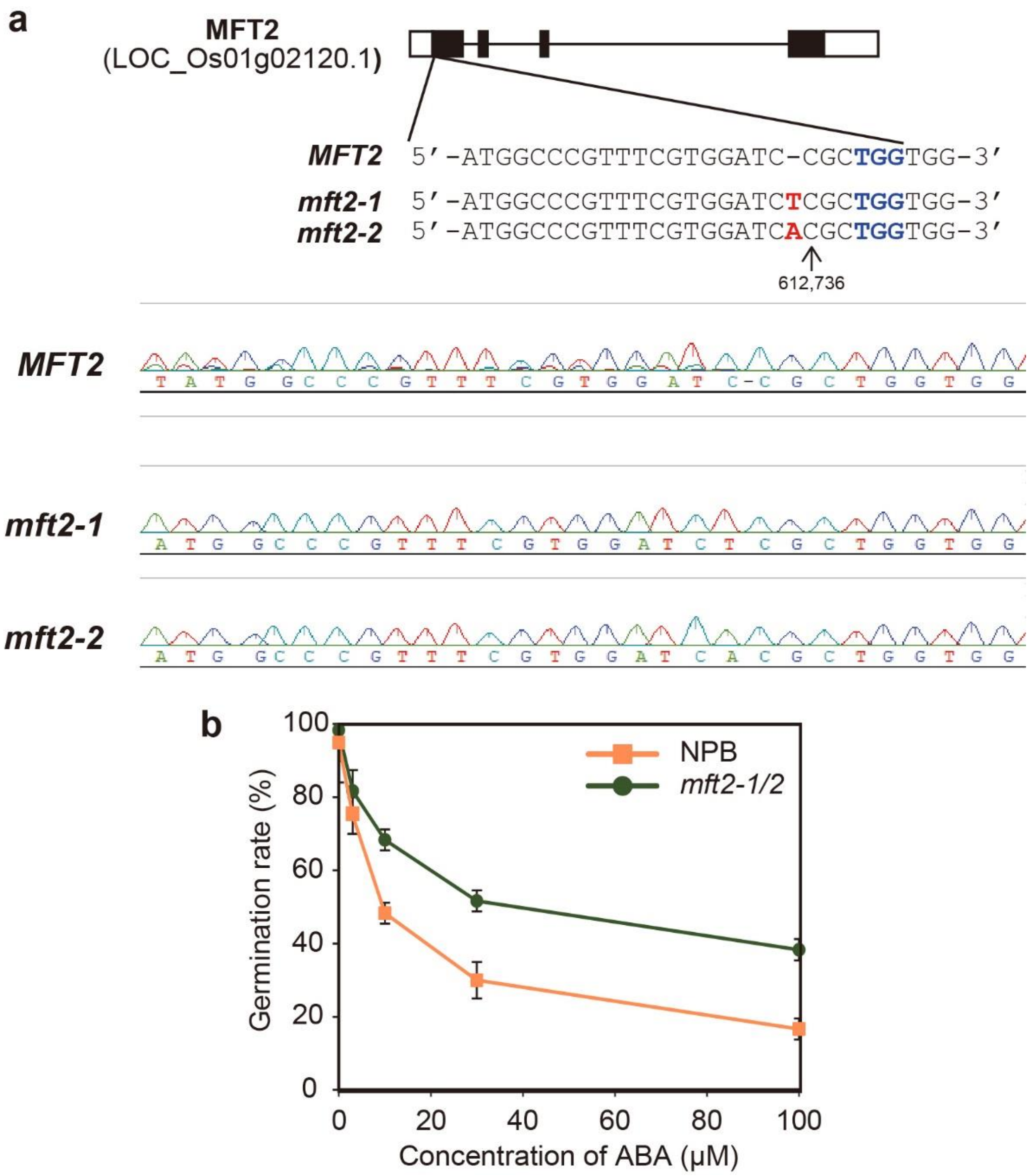

Supplementary Fig. 14. ABA response of the MFT2 knockout mutant. a, Diagram of MFT2 CRISPR knockout lines (*mft2-1* and *mft2-2*). **b**, ABA response of *mft2* and control plants, Nipponbare (NPB). The centre for the error bars represents mean. Error bars, s.d. (n = 3 biologically independent samples). Source data are provided as a Source Data file.

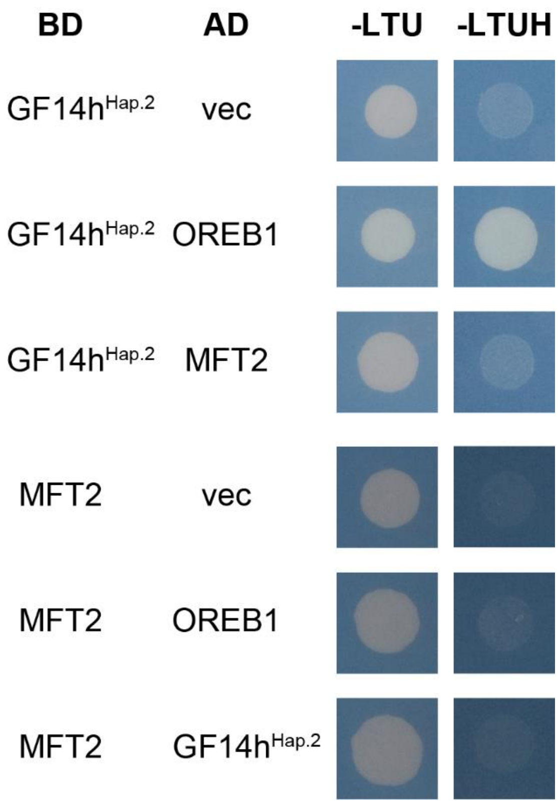

Supplementary Fig. 15. Interactions between GF14h$^{Hap.2}$, OREB1, and MFT2 in a Y2H assay. AD, GAL4-activating domain; BD, GAL4-binding domain; −LTU, synthetic complete medium lacking Leu, Trp, and Ura; −LTUH, synthetic complete medium lacking Leu, Trp, Ura, and His.

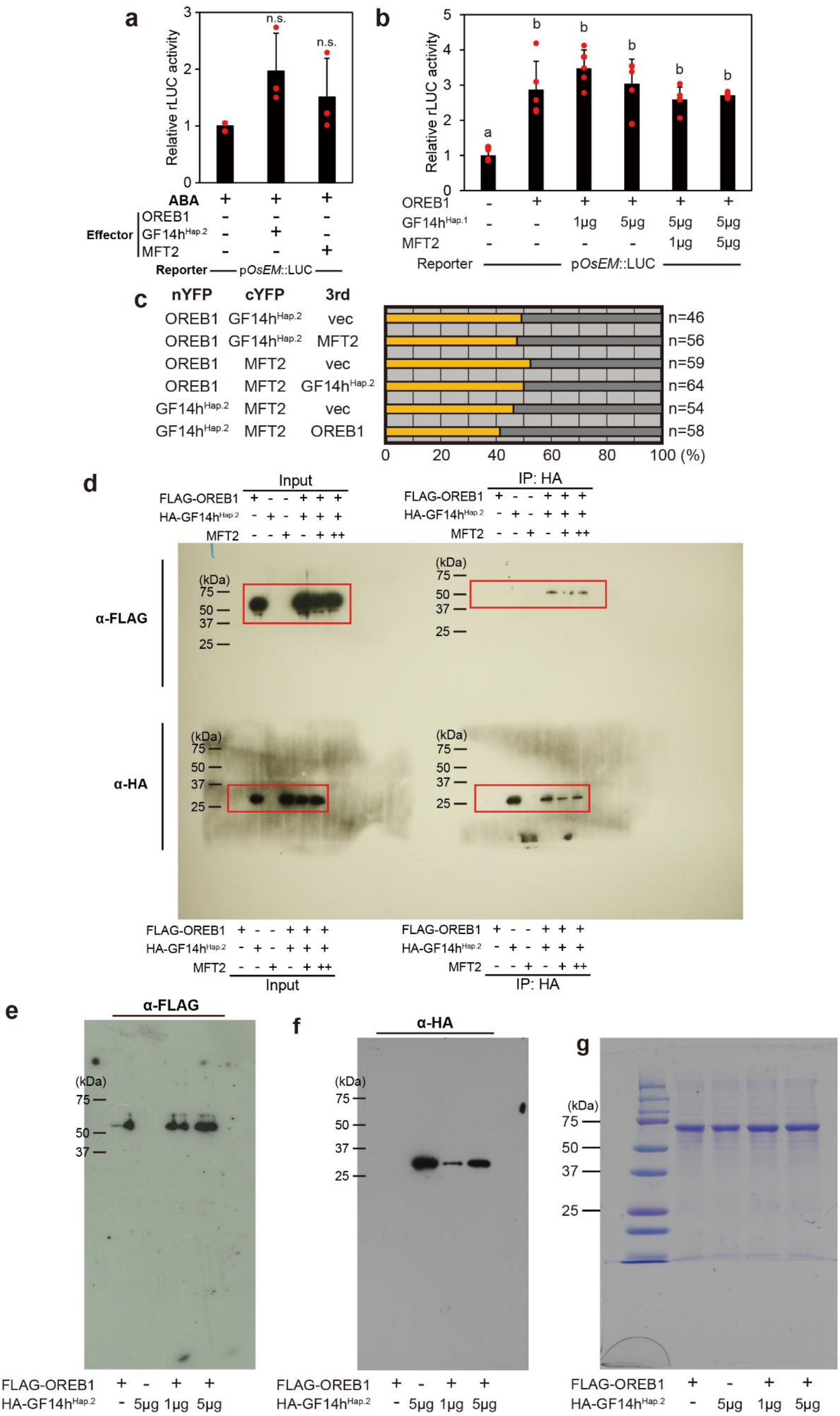
a
Relative rLUC activity
n.s.
ABA
Effector
OREB1
GF14h^Hap.2
MFT2
Reporter
pOsEM::LUC
b
Relative rLUC activity
OREB1
GF14h^Hap.1
MFT2
1μg
5μg
Reporter
pOsEM::LUC
c
nYFP
cYFP
3rd
OREB1
GF14h^Hap.2
vec
MFT2
n=46
n=56
n=59
n=64
n=54
n=58
100 (%)
d
Input
IP: HA
FLAG-OREB1
HA-GF14h^Hap.2
MFT2
α-FLAG
α-HA
(kDa)
e
α-FLAG
f
α-HA
g
FLAG-OREB1
HA-GF14h^Hap.2
5μg
1μg

Supplementary Fig. 16. Relationship between GF14h, OREB1, and MFT2. **a**, Transient reporter assay to examine the transactivation effect of GF14h$^{Hap.2}$ and MFT2 on the expression of *OsEM* without OREB1. The activity by vector control (left) was set to 1. n.s. indicates no significant difference compared to the vector control by two-sided student's t-test. The centre for the error bars represents mean. Error bars, s.d. (n = 3 biologically independent samples). **b**, Transient reporter assay to examine the transactivation effect of GF14h$^{Hap.1}$ on the expression of *OsEM*. The activity by vector control (left) was set to 1. Letters indicate significant differences (P <0.01, Tukey's HSD test). The centre for the error bars represents mean. Error bars, s.d. (n >4 biologically independent samples). The exact *P*-values were shown in Source Data file. **c**, Quantification of the BiFC assays is shown in Fig. 4f. Effects of the non-tagged 3rd factors were examined. **d**. Uncropped scans of gel blotting are shown in Fig. 4h. **e,f**, Uncropped scans of gel blotting for the protein extracted from the protoplasts introduced with FLAG-OREB1 and HA-GF14h$^{Hap.2}$ as described with anti-FLAG (**e**) or anti-HA (**f**). **g,** Uncropped scans of the CBB staining of the protein samples used in e and f. For **e-g**, each experiment was repeated independently for at least 3 times with similar results. Source data are provided as a Source Data file.

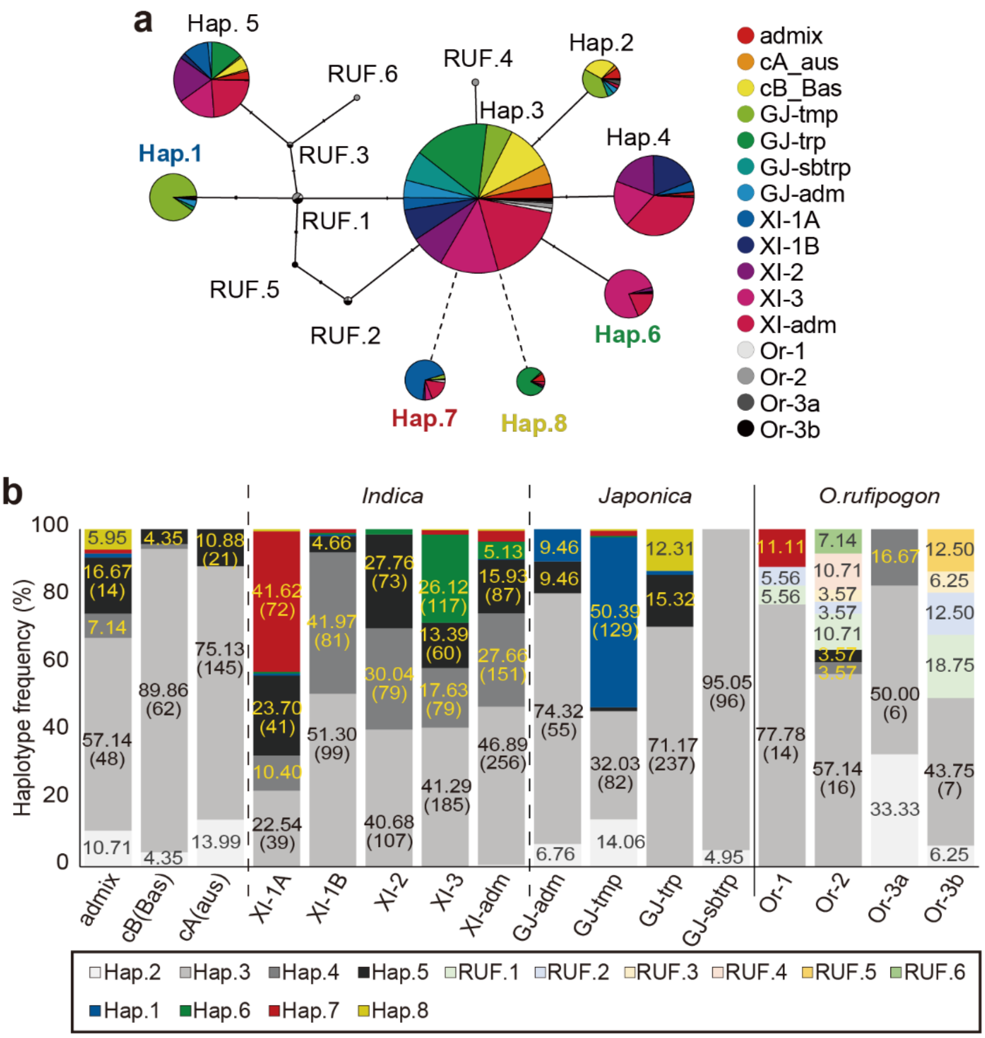

Supplementary Fig. 17. Haplotype frequency of *GF14h*. a, Haplotype network analysis of *GF14h* using the genotype data of 2733 *Oryza sativa* and 74 *Oryza rufipogon* accessions. **b**, Haplotype frequency in different ecotypes of the 2807 rice accessions in the 3K panel and 74 *O. rufipogon*. Numbers in bars represent the percentage of haplotypes and the number of accessions in parentheses.

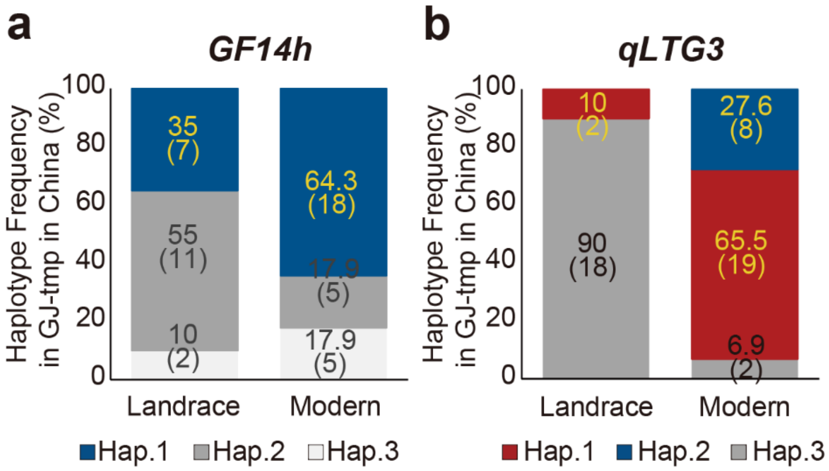

Supplementary Fig. 18. Domestication of GF14h and qLTG3 in China. a,**b**, Haplotype frequency for GF14h (a) and qLTG3 (b) in Chinese temperate *japonica* accessions of the 3K panel.

Supplementary Table 1. The detail information of the polymorphism in Peak 1 for G × E GWAS shown in Fig. 1d.

chrom	pos	-log10(p)	effect@30	effect@15	effect.std@30	effect.std@15	wald@30	wald@15	MAF
11	23420427	8.42217	0.5863428	0.3949303	0.444895839	0.369420662	9.17479	1.23734	0.16312
11	23555223	6.11207	0.544094	0.3612135	0.448698271	0.373316641	4.31484	0.27057	0.13245

Supplementary Table 2. The list of 74 wild rice accessions (*O. rufipogon*).

Serial number	Accession ID	Ecotype	Original producing area	Latitude	Longitude	Sequence Depth	Origin of the Fastq file	GF14h_Hap	qLTG3_Hap
1	W0106	Or-I	India	20.46	85.88	12.14	d	Hap.3	Hap.3
2	W0120	Or-II	India	20.46	85.88	32.48	d	RUF.6	Hap.4
3	W0123	Or-I	India	20.46	85.93	38.66	b	Hap.3	Hap.3
4	W0128	Or-I	India	21	85.1	84.57	b	Hap.3	Hap.4
5	W0137	Or-IIIb	India	16.91	81.82	28.02	d	RUF.2	Hap.3
6	W0141	Or-IIIb	India	10.37	76.37	75.79	b	RUF.5	Hap.3
7	W0163	Or-II	Thailand	18.81	98.66	2.94	a	RUF.6	NA
8	W0170	Or-I	Thailand	15.87	100.99	78.34	b	Hap.3	Hap.3
9	W0171	Or-II	Thailand	15.87	100.99	5.34	a	Hap.3	Hap.3
10	W0175	Or-II	Thailand	18.08	103.27	3.24	a	Hap.4	Hap.4
11	W0180	Or-II	Thailand	18.77	99.97	29.36	d	Hap.3	Hap.3
12	W0576	Or-II	Malaya	5.8	102.38	3.25	a	RUF.4	Hap.3
13	W0593	Or-IIIb	Malaya	3.14	101.69	33.52	d	Hap.3	Hap.3
14	W0630	Or-I	Burma	20.46	94.56	37.35	d	Hap.3	Hap.3
15	W1087	Or-II	India	26.15	91.74	5.34	a	Hap.3	NA
16	W1096	Or-II	India	26.2	92.94	4	a	Hap.3	Hap.3
17	W1230	Or-I	Dutch New Guinea	-4.63	138.93	32.4	d	Hap.3	Hap.3
18	W1236	Or-II	Australian New Guinea	-5.31	141.61	33.42	d	RUF.3	Hap.4
19	W1294	Or-IIIb	Philippines	7.86	124.86	11.13	d	RUF.1	Hap.3
20	W1551	Or-I	Thailand	14.5	100.89	14.17	d	Hap.3	Hap.3
21	W1554	Or-II	Thailand	15.09	99.99	2.74	a	Hap.3	NA
22	W1669	Or-IIIb	India	19.08	82.45	13.01	d	RUF.3	Hap.3
23	W1681	Or-I	India	20.09	84.45	16.39	d	RUF.2	Hap.3
24	W1683	Or-II	India	20.1	84.48	5.86	a	RUF.2	NA
25	W1687	Or-II	India	23.04	88.17	61.57	b	Hap.3	Hap.3
26	W1698	Or-I	Thailand	14.34	100.59	63.19	b	Hap.3	Hap.3
27	W1718	Or-II	China	NA	NA	7.28	c	RUF.4	Hap.3
28	W1739	Or-IIIb	India	26.92	75.82	99.51	b	RUF.1	Hap.3
29	W1754	Or-I	India	20.27	81.5	106.85	b	RUF.1	Hap.3
30	W1777	Or-IIIb	India	19.95	79.3	114.48	b	RUF.2	Hap.3
31	W1782	Or-IIIb	India	12.31	76.64	3.24	a	Hap.3	NA
32	W1790	Or-II	Thailand	15	100	6.21	a	RUF.1	Hap.4
33	W1807	Or-IIIb	Sri Lanka	6.93	79.95	19.7	d	Hap.3	Hap.4
34	W1849	Or-II	Thailand	19.56	99.7	9.04	a	Hap.5	Hap.3
35	W1854	Or-II	Thailand	19.64	99.52	4.12	a	Hap.3	NA
36	W1859	Or-II	Thailand	17.52	100.12	4.12	a	Hap.3	NA
37	W1866	Or-I	Thailand	14.57	100.99	19.07	d	Hap.3	Hap.3
38	W1886	Or-I	Thailand	NA	NA	20.72	d	Hap.3	Hap.3
39	W1921	Or-I	Thailand	14.44	100.9	11.17	d	Hap.3	Hap.3
40	W1940	Or-II	Thailand	14.92	103.51	4.63	a	Hap.3	NA
41	W1943	Or-IIIa	China	NA	NA	163.65	b	Hap.2	Hap.3
42	W1944	Or-IIIa	NA	NA	NA	7.79	c	Hap.4	Hap.3
43	W1945	Or-IIIa	China	NA	NA	14.92	d	Hap.2	Hap.3
44	W1962	Or-IIIa	China	NA	NA	15.7	d	Hap.2	Hap.3
45	W1977	Or-II	Indonesia	-6.4	106.82	3.39	a	RUF.4	NA
46	W1979	Or-II	Indonesia	-6.4	106.82	56.41	b	Hap.3	Hap.3
47	W1981	Or-II	Indonesia	-2.99	104.76	32.3	d	Hap.3	Hap.3
48	W2003	Or-IIIb	India	15.3	73.5	20.15	d	RUF.5	Hap.3
49	W2012	Or-IIIb	India	19.8	72.55	103.07	b	RUF.1	Hap.3
50	W2022	Or-II	Indonesia	3.29	117	3.85	a	Hap.3	NA
51	W2024	Or-II	Indonesia	3.29	117	3.89	a	Hap.3	NA
52	W2051	Or-II	Bangladesh	23.71	90.41	14.87	d	Hap.3	Hap.4
53	W2057	Or-II	Bangladesh	24.48	91.78	25.15	d	RUF.1	Hap.3
54	W2109	Or-II	Australia	NA	NA	15.43	d	RUF.1	Hap.3
55	W2114	Or-IIIb	Australia	NA	NA	16.01	d	Hap.3	Hap.3
56	W2117	Or-IIIb	Australia	NA	NA	11.78	d	Hap.3	Hap.3
57	W2198	Or-IIIa	China	NA	NA	4.08	a	Hap.3	NA
58	W2263	Or-I	Cambodia	11.33	104.5	15.65	d	Hap.3	Hap.3
59	W2282	Or-II	Thailand	16.49	99.47	2.87	a	Hap.3	NA
60	W2306	Or-I	Laos	14.5	105.5	2.86	a	Hap.3	Hap.3
61	W2316	Or-I	Vietnam	10.39	107.02	3.4	a	Hap.7	NA
62	W2318	Or-II	Vietnam	10.24	106.06	3.09	a	Hap.3	NA
63	W2319	Or-I	Vietnam	10.33	106.25	2.71	a	Hap.7	Hap.3
64	W3014	Or-IIIa	China	23.29	113.83	3.2	a	Hap.3	NA
65	W3027	Or-IIIa	China	21.95	108.61	3.5	a	Hap.4	NA
66	W3044	Or-IIIa	China	23.17	108.28	3.38	a	Hap.3	NA
67	W3046	Or-IIIa	China	22.69	109.27	4.19	a	Hap.3	Hap.3
68	W3048	Or-IIIa	China	23.9	106.61	3.85	a	Hap.3	Hap.3
69	W3065	Or-IIIb	China	19.25	110.46	3.54	a	Hap.3	Hap.3
70	W3070	Or-IIIb	China	18.65	109.8	3.81	a	Hap.2	NA
71	W3071	Or-IIIb	China	19.62	110.7	2.83	a	Hap.3	NA
72	W3078	Or-IIIa	China	28.23	116.61	90.19	b	Hap.3	Hap.3
73	W3095	Or-IIIa	China	25.28	111.34	93.25	b	Hap.2	Hap.3
74	W3105	Or-I	India	NA	NA	152.09	b	Hap.3	Hap.4

(a) Huang, X. et al. 2012
(b) Zhao Q. et al. 2018
(c) Our Lab (PRJNA674962)
(d) National Institute of Genetics, Japan

Supplementary Table 3. Haplotype analysis of *GF14h* using the 2733 *O. sativa* accessions and 74 *O. rufipogon* accessions.

REF A.A	GAG(E)	GTT(V)	GGA(G)	GTG(V)	-	GCC(A)	GAG(E)	GCC(A)	-	AAT(N)	GAC(D)	ACA(T)	functional(+) non-functional(-)																		
Position of A.A	+247	+244	+235	+209	+179	+164	+160	+157	+143	+111	+74	+71																			
ALT A.A	GAC(D)	ATT(I)	GAA(E)	TTG(L)	4bp in	GTC(V)	GAC(D)	TCC(S)	1bp in	AGT(S)	GAG(E)	ATA(I)																			
REF allele	C	C	C	C	C	G	C	C	C	T	G	G		Total		admix	cB(Bas)	cA(aus)	XI-1A	XI-1B	XI-2	XI-3	XI-adm	GJ-adm	GJ-tmp	GJ-trp	GJ-sbtrp	Or-I	Or-II	Or-IIIa	Or-IIIb
Position (IRGSP1.0)	23553901	23553912	23554688	23554767	23555223	23555269	23555280	23555291	23555426	23556806	23556916	23556926																			
ALT allele	G	T	T	A	CATAA	A	G	A	CA	C	C	A																			
Hap.1	C	C	C	C	C	G	C	C	C	T	G	G	-	144	Hap.1	1	0	0	1	1	0	0	1	7	129	4	0	0	0	0	0
Hap.2	C	C	C	C	CATAA	A	C	A	C	T	C	G	+	93	Hap.2	9	3	27	0	0	0	0	3	5	36	0	5	0	0	4	1
Hap.3	C	C	C	C	CATAA	G	C	A	C	T	C	G	+	1454	Hap.3	48	62	145	39	99	107	185	256	55	82	237	96	14	16	6	7
Hap.4	C	T	C	C	CATAA	G	C	A	C	T	C	G	+	418	Hap.4	6	1	0	18	81	79	79	151	0	0	0	0	0	1	2	0
Hap.5	G	C	T	C	CATAA	G	C	C	C	T	C	G	+	370	Hap.5	14	3	21	41	9	73	60	87	7	3	51	0	0	1	0	0
Hap.6	C	C	C	C	CATAA	G	C	A	CA	T	C	G	-	152	Hap.6	0	0	0	1	1	4	117	28	0	1	0	0	0	0	0	0
Hap.7	NA	NA	NA	NA	NA	NA	NA	NA	NA	NA	NA	NA	-	105	Hap.7	1	0	0	72	2	0	6	18	0	4	0	0	2	0	0	0
Hap.8	NA	NA	NA	NA	NA	NA	NA	NA	NA	T	C	G	-	51	Hap.8	5	0	0	1	0	0	1	2	0	1	41	0	0	0	0	0
RUF.1	C	C	C	C	CATAA	G	C	C	C	T	C	G	+	7	RUF.1	0	0	0	0	0	0	0	0	0	0	0	0	1	3	0	3
RUF.2	C	C	C	C	CATAA	G	C	A	C	C	C	G	+	4	RUF.2	0	0	0	0	0	0	0	0	0	0	0	0	1	1	0	2
RUF.3	G	C	C	C	CATAA	G	C	C	C	T	C	G	+	2	RUF.3	0	0	0	0	0	0	0	0	0	0	0	0	0	1	0	1
RUF.4	C	C	C	A	CATAA	G	C	A	C	T	C	G	+	3	RUF.4	0	0	0	0	0	0	0	0	0	0	0	0	0	3	0	0
RUF.5	C	C	C	C	CATAA	G	G	C	C	C	C	G	+	2	RUF.5	0	0	0	0	0	0	0	0	0	0	0	0	0	0	0	2
RUF.6	G	C	C	C	CATAA	G	C	C	C	T	C	A	+	2	RUF.6	0	0	0	0	0	0	0	0	0	0	0	0	0	2	0	0

Supplementary Table 4. Haplotype analysis of *qLTG3* using the 2875 *O. sativa* accessions and 54 *O. rufipogon* accessions.

REF A.A	'	CAC(H)	TCA(S)	GOF(+) partially LOF(-) LOF(--)																	
Position of A.A	+16	+17	+62																		
ALT A.A	71bp del	CTC(L)	GGA(G)																		
REF allele	*	A	TC		total	admix	cA(aus)	cB(Bas)	XI-1A	XI-1B	XI-2	XI-3	XI-adm	GJ-adm	GJ-tmp	GJ-trp	GJ-sbtrp	Or- I	Or- II	Or- IIIa	Or- IIIb
Position (IRGSP1.0)	220111	220116	220249																		
ALT allele	C	T	GG																		
Hap.1	*	A	TC	-	148	8	0	0	0	6	0	0	5	14	112	3	0	0	0	0	0
Hap.2	C	Deletion	TC	--	37	1	0	0	0	1	0	0	2	0	33	0	0	0	0	0	0
Hap.3	*	T	TC	+	1325	54	67	90	78	78	133	5	125	59	116	362	112	15	11	8	12
Hap.4	*	T	GG	+	1419	23	3	106	121	113	142	454	448	1	0	0	0	2	5	0	1

*CCTCCACTTCTTCACCTTCTCCGACGCGTGCGGCTGCCAGTGCGCTCATGCCCTAG TCCCGGCGGAGGAGG

Supplementary Table 5. Primers used in this study.

Experiments	Primer name	Sequence
Complementation assay	14_3_3_genome_U	caccGGGTAAGTGGTACTCATTGAATCAT
	14_3_3_genome_L	CAATGTTACTGATTGTTTTGCTCAC
Transgenic experiment	14_3_3_ox_U	caccATGAAGGAGAGGGAGAAGGT
	14_3_3_ox_L	TTAGCCCTCCATAACAACAT
MFT2 CRISPR	OsMFT2_guide1_F	gttgGATCGTGGTTAACATACCGG
	OsMFT2_guide1_R	aaacCCGGTATGTTAACCACGATC
	OsMFT2_check_F	ATAGCATGATCGAGTGAGTGG
	OsMFT2_check_R1	CAGGGTGTAGAGGTCGTTGCG
GF14h promoter-GUS	14_3_3_GFP_U	caccGGGTAAGTGGTACTCATTGAATCAT
	14_3_3_GFP_L	CTTCTTCACTTCCCAGCAATGGATT
Yeast two hybrid assay	GF14-H-EcoRI_F1	GGAATTCATGAAGGAGAGGGAGAAGGT
	GF14-H-SmaI_R1	TCCCCCGGGTTAGCCCTCCATAACAACAT
	OREB1-SmaI_F1	TCCCCCGGGATGGCATCGGAGATGAGC
	OREB1-BamHI_R1	GCGGATCCTCACCACATGCAGCTGC
	TRAB1-EcoRI_F1	GGAATTCATGAACATGGACGAGCTGC
	TRAB1-SmaI_R1	TCCCCCGGGTTACCAGGGACCTGTCAATG
	cacc+GF14h_F	caccATGAAGGAGAGGGAGAAGGTGG
	GF14h_R	TTAGCCCTCCATAACAACATCATCGTC
	cacc+MFT2_F	caccATGGCCCGTTTCGTGGATCCGC
	MFT2_R	TTAGCGGCGGCGGTTGGCGGGCTCCC
	cacc+OREB1_F	caccATGGCATCGGAGATGAGCAAGAAC
	OREB1_R	TCACCACATGCAGCTGCCGCTGCGGCG
Subcellular localisation	NEBuilder_pE2113_p35s_F	ggccagtgccaagctttgagacttttcaacaaag
	NEBuilder_OREB1_GFP_R	cttgctcaccatactagtCCACATGCAGCTGCCG
	NEBuilder_OREB1(S385E)_GFP_R	cttgctcaccatactagtCCACATGCACTCGCCGCT
	NEBuilder_GF14h_Hap1_GFP_R	cttgctcaccatactagtTCTCAAGAAGACTGAAAAG
	NEBuilder_GF14h_GFP_R	cttgctcaccatactagtGCCCTCCATAACAACATC
	NEBuilder_MFT2_GFP_R	cttgctcaccatactagtGCGGCGGCGGTTGGC
	pE2113_n_mSCARLET.F	TACATCTAGGACTAGTATGGTATCAAAGGGAGAGG
	pE2113_n_mSCARLET.R	GCTCTCTAGAAGGCCTCTTATACAACTCATCCATA
	pE2113_GW_mSCARLET.F	TAACAGCGCTACTAGTATGGTATCAAAGGGAGAGG
	pE2113_GW_mSCARLET.1.R	TCGAGCTCTAAGGCCTTCACTTATACAACTCATCC
	OsNST1.mRNA.F	ATATCCTCGTGACTTCTCCTCCTCCTGAGT
	OsNST1.mRNA.R	TGCCTACTGCAAGATCAAAGGGAAGTAG
	OsNST1.pE2113mSC.F	TACAACTACATCTAGAATGGCGAAGGGAGGGGGG
	OsNST1.pE2113mSC.R	TTGATACCATACTAGTCTTCCCTTTGATCTTGCAG
	OsPUP1.const.F	TAACTACCATCAACACACCCTAATC
	OsPUP1.const.R.true	AAACATGTTCACTACACCCGTAAAT
	OsPUP1.n.mSC.F	TAAGAGGCCTTCTAGAATGGCCACCATTACTGCTG
	PUP.mSC.2.R	GATCGGGGAAATTCGAGCTCCTAAGGCGCCGCTGACTC
	OsIDD2.mSC.F	tacaactacatctagaATGATGCTCAAGGATCTGG
	OsIDD2.mSC.R	ttgataccatactagtGCTATGCCATGGCTTGTTG
BiFC assay	cacc+GF14h_F	caccATGAAGGAGAGGGAGAAGGTGG
	GF14h_nonstop_R	GCCCTCCATAACAACATCATCGTCCTTA
	GF14h_R	TTAGCCCTCCATAACAACATCATCGTC
	cacc+MFT2_F	caccATGGCCCGTTTCGTGGATCCGC
	MFT2_nonstop_R	TTAGCGGCGGCGGTTGGCGGGCTCCC
	MFT2_R	GCGGCGGCGGTTGGCGGGCTCCCTC
	cacc+OREB1_F	caccATGGCATCGGAGATGAGCAAGAAC
	OREB1_nonstop_R	CCACATGCAGCTGCCGCTGCGGCGCAG
	OREB1_R	TCACCACATGCAGCTGCCGCTGCGGCG
	cacc+GF14h_F	caccATGAAGGAGAGGGAGAAGGTGG
	GF14h_Hap1_nonstop_R	TCTCAAGAAGACTGAAAAGTTGAGCGC
	GF14h_Hap1_R	TTATCTCAAGAAGACTGAAAAGTTGAG
Point mutation	OREB1_S385A_F2	CAGCTGCGCCGCAGCGGCGCCTGCATGTGG
	OREB1_S385A_R2	CCACATGCAGGCGCCGCTGCGGCGCAGCTG
	OREB1(S385E)_point_mutation_F	CAGCTGCGCCGCAGCGGCGAGTGCATGTGG
	OREB1(S385E)_nonstop_R	CCACATGCACTCGCCGCTGCGGCGCAGCTG
Transient reporter assay	HindIII+OsEM_F	CCCAAGCTTatacacatgcagttgcacaca
	OsEM_R+SmaI	TCCCCCGGGtgccaaacactagttagctag
	XbaI+GF14h.inf.F	TACAACTACATCTAGAATGAAGGAGAGGGAGAAGG
	GF14h+SpeI.inf.R	GCCTGGGCCCACTAGTTTAGCCCTCCATAACAACAT
	XbaI+OREB1.inf.F	TACAACTACATCTAGAATGGCATCGGAGATGAGC
	OREB1+SpeI.inf.R	GCCTGGGCCCACTAGTTCACCACATGCAGCTGCC
	XbaI+MFT2.inf.F	TACAACTACATCTAGAATGGCCCGTTTCGTGGAT
	MFT2+SpeI.inf.R	GCCTGGGCCCACTAGTTTAGCGGCGGCGGTTGGC
	NEB_omega-GF14h_F	tagactagaggatccATGAAGGAGAGGGAG
	NEB_GF14h-Hap1_nosT_R	agctcggtacccgggTTATCTCAAGAAGAC
Co-immunoprecipitation	SpeI_sGFP_StuI_XbaI_SacI_OFF.F	TACAACTACATCTAGgACTAGTATGGTGAGCAAGG
	SpeI_sGFP_StuI_XbaI_SacI_++ON.R	GATCGGGGAAATTCGAGCTCTCTAGAAGGCCTcttgtacagctcg
	SpeI+3xFLAG.F	gactagtgattacaaggatgacgacgataa
	3xFLAG.R+StuI	ccaaggccttttatcgtcatcatctttgtaat
	OREB1.pnFL.F	TAAAAGGCCTTCTAGAATGGCATCGGAGATGAGC
	OREB1.pnFL.R	GATCGGGGAAATTCGAGCTCTCACCACATGCACTCGCC
	NEBuilder_HA_pE2113_F	caactacatctaggaATGGAGTACCCATACGACGTAC
	NEBuilder_GF14h_pE2113_R	tgaacgatcggggaaattcgTTAGCCCTCCATAACAAC
	NEBuilder_MYC_pE2113_F	caactacatctaggaATGGAGGAGCAGAAGCTGATCTC
	NEBuilder_MFT2_pE2113_R	tgaacgatcggggaaattcgTTAGCGGCGGCGGTTGGC
	NEBuilder_OREB1_F	TCTACAACTACATCTAGAATGGCATCGGAGATGAGC
	NEBuilder_OREB1_3xFLAG_R	TCCTTGTAATCACTAGTCCACATGCAGCTGCCGCT

Supplementary references

1. Yano, K. *et al.* Genome-wide association study using whole-genome sequencing rapidly identifies new genes influencing agronomic traits in rice. *Nat Genet* **48**, 927–934 (2016).
2. Taoka, K. *et al.* 14-3-3 proteins act as intracellular receptors for rice Hd3a florigen. *Nature* **476**, 332–335 (2011).
