# Supplementary material for: Genome-wide association study identifies a gene responsible for temperature-dependent rice germination
Source: Nat Commun. 2022 Sep 29;13:5665. doi: 10.1038/s41467-022-33318-5 (PMC9523024; doi:10.1038/s41467-022-33318-5)
Supplement: Supplementary file 3 — Description of Additional Supplementary Files [file 41467_2022_33318_MOESM3_ESM.pdf]

Description of Additional Supplementary Files

File Name: Supplementary Data 1

Description: List of the 164 rice varieties used in this study.

File Name: Supplementary Data 2

Description: BLUP estimation of allelic effects for G × E GWAS shown in Fig. 1a.

File Name: Supplementary Data 3

Description: BLUP estimation of allelic effects for G × E GWAS shown in Fig. 1d.
