# Supplementary material for: Genome-wide association study identifies a gene responsible for temperature-dependent rice germination
Source: Nat Commun. 2022 Sep 29;13:5665. doi: 10.1038/s41467-022-33318-5 (PMC9523024; doi:10.1038/s41467-022-33318-5)
Supplement: Supplementary file 7 — Reporting Summary [file 41467_2022_33318_MOESM7_ESM.pdf]

nature portfolio

Corresponding author(s): Hideki Yoshida, Makoto Matsuoka

Last updated by author(s): Jul 19, 2022

Reporting Summary

Nature Portfolio wishes to improve the reproducibility of the work that we publish. This form provides structure for consistency and transparency in reporting. For further information on Nature Portfolio policies, see our Editorial Policies and the Editorial Policy Checklist.

Statistics

For all statistical analyses, confirm that the following items are present in the figure legend, table legend, main text, or Methods section.

n/a	Confirmed	
☐	☒	The exact sample size (*n*) for each experimental group/condition, given as a discrete number and unit of measurement
☐	☒	A statement on whether measurements were taken from distinct samples or whether the same sample was measured repeatedly
☐	☒	The statistical test(s) used AND whether they are one- or two-sided<br>*Only common tests should be described solely by name; describe more complex techniques in the Methods section.*
☒	☐	A description of all covariates tested
☒	☐	A description of any assumptions or corrections, such as tests of normality and adjustment for multiple comparisons
☐	☒	A full description of the statistical parameters including central tendency (e.g. means) or other basic estimates (e.g. regression coefficient) AND variation (e.g. standard deviation) or associated estimates of uncertainty (e.g. confidence intervals)
☐	☒	For null hypothesis testing, the test statistic (e.g. *F*, *t*, *r*) with confidence intervals, effect sizes, degrees of freedom and *P* value noted<br>*Give P values as exact values whenever suitable.*
☐	☒	For Bayesian analysis, information on the choice of priors and Markov chain Monte Carlo settings
☒	☐	For hierarchical and complex designs, identification of the appropriate level for tests and full reporting of outcomes
☒	☐	Estimates of effect sizes (e.g. Cohen's *d*, Pearson's *r*), indicating how they were calculated

Our web collection on statistics for biologists contains articles on many of the points above.

Software and code

Policy information about availability of computer code

Data collection	ABI3730, ZEN 2.3 version 14.0.12.201, CFX Maestro v1.0
Data analysis	R:v4.0.3, samtools:v1.10, bwa:v0.7.17-r1198-dirty, vcftools:v0.1.17, PopART:v1.7, MrBayes:v3.2.7a

For manuscripts utilizing custom algorithms or software that are central to the research but not yet described in published literature, software must be made available to editors and reviewers. We strongly encourage code deposition in a community repository (e.g. GitHub). See the Nature Portfolio guidelines for submitting code & software for further information.

Data

Policy information about availability of data

All manuscripts must include a data availability statement. This statement should provide the following information, where applicable:

- Accession codes, unique identifiers, or web links for publicly available datasets
- A description of any restrictions on data availability
- For clinical datasets or third party data, please ensure that the statement adheres to our policy

The sequence data has been deposited in DNA Data Bank of Japan Sequence Read Archive (DRA): DRA004358, DRA008452 and DRA012529.

Human research participants

Policy information about studies involving human research participants and Sex and Gender in Research.

Reporting on sex and gender	not applicable
Population characteristics	not applicable
Recruitment	not applicable
Ethics oversight	not applicable

Note that full information on the approval of the study protocol must also be provided in the manuscript.

Field-specific reporting

Please select the one below that is the best fit for your research. If you are not sure, read the appropriate sections before making your selection.

- [x] Life sciences
- [ ] Behavioural & social sciences
- [ ] Ecological, evolutionary & environmental sciences

For a reference copy of the document with all sections, see nature.com/documents/nr-reporting-summary-flat.pdf

Life sciences study design

All studies must disclose on these points even when the disclosure is negative.

Sample size	Sample sizes were determined based on previous reports and best practices.
Data exclusions	No data were excluded from the analyses.
Replication	More than three replicates were taken for the measurements, all attempts at replication were successful.
Randomization	Due to the nature of the experimental setup blinding/randomisation was not practical.
Blinding	Due to the nature of the experimental setup blinding/randomisation was not practical.

Reporting for specific materials, systems and methods

We require information from authors about some types of materials, experimental systems and methods used in many studies. Here, indicate whether each material, system or method listed is relevant to your study. If you are not sure if a list item applies to your research, read the appropriate section before selecting a response.

Materials & experimental systems

n/a	Involved in the study	
[ ]	[x]	Antibodies
[ ]	[x]	Eukaryotic cell lines
[x]	[ ]	Palaeontology and archaeology
[x]	[ ]	Animals and other organisms
[x]	[ ]	Clinical data
[x]	[ ]	Dual use research of concern

Methods

n/a	Involved in the study	
[x]	[ ]	ChIP-seq
[x]	[ ]	Flow cytometry
[x]	[ ]	MRI-based neuroimaging

Antibodies

Antibodies used	Anti-FLAG M2 (Sigma-Aldrich; F3165), Anti-HA (Sigma; H9658), anti-FLAG M2-HRP (Sigma; A8592), anti-HA-HRP antibodies (Sigma; H6533)
Validation	Anti Flag M2 antibody is used for the detection of Flag fusion proteins.This monoclonal antibody is produced in mouse and recognizes the FLAG sequence at the N-terminus, Met N-terminus, and C-terminus. The antibody is also able to recognize FLAG at an internal site. M2, unlike M1 antibody is not Calcium dependent.<br><br>Anti-HA recognizes native as well as denatured-reduced forms of HA-tagged proteins and is reactive with N- or C-terminal HA-tagged fusion proteins.

The Monoclonal ANTI-FLAG M2-Peroxidase is a mouse IgG antibody covalently conjugated to horseradish peroxidase (HRP). The antibody binds to FLAG fusion proteins and recognizes the FLAG epitope at N-terminal, Met-N-terminal, C-terminal, and internal FLAG peptides.

ATAnti-HA–HRP recognizes native as well as denatured-reduced forms of HA-tagged proteins and is reactive with N- or C-terminal HA-tagged fusion proteins.

Eukaryotic cell lines

Policy information about cell lines and Sex and Gender in Research

Cell line source(s)	Yeast strains (Y187 or PJ69-4A) from National BioResource Project (NBRP).
Authentication	Yeast strains were verified by NBRP (Y187; https://yeast.nig.ac.jp/yeast/by/StrainDetail.jsf?id=3912, PJ69-4A; https://yeast.nig.ac.jp/yeast/by/StrainDetail.jsf?id=20246)
Mycoplasma contamination	The yeast strain was not tested for mycoplasma contamination.
Commonly misidentified lines (See ICLAC register)	No commonly misidentified lines are used in this study.
